# Solid Phase *versus* Solution Phase Synthesis of Heterocyclic Macrocycles

**DOI:** 10.3390/molecules18011111

**Published:** 2013-01-16

**Authors:** Seong Jong Kim, Shelli R. McAlpine

**Affiliations:** School of Chemistry, University of New South Wales, Sydney, NSW 2052, Australia

**Keywords:** urukthapelstatin A, cytotoxic, macrocycle, peptides, heterocycle, thiazole, oxazole, telomestatin, solid phase peptide synthesis

## Abstract

Comparing a solution phase route to a solid phase route in the synthesis of the cytotoxic natural product urukthapelstatin A (Ustat A) confirmed that a solid phase method is superior. The solution phase approach was tedious and involved cyclization of a ridged heterocyclic precursor, while solid phase allowed the rapid generation of a flexible linear peptide. Cyclization of the linear peptide was facile and subsequent generation of three oxazoles located within the structure of Ustat A proved relatively straightforward. Given the ease with which the oxazole Ustat A precursor is formed via our solid phase approach, this route is amenable to rapid analog synthesis.

## 1. Introduction

Natural product-based macrocyclic peptides have tremendous potential as drug leads [1,2,3,4,5]. They have numerous pharmacokinetic benefits as drug candidates, including metabolic stability, structural rigidity, high affinity to biological targets and reasonable solubility properties [6,7]. As of 2009, there were 617 peptide drugs or drug candidates, 24% of these are in clinical trials, 65% are in advanced preclinical phases, and 11% are on the market [3]. These peptide drugs are used to treat numerous diseased states including: prostate and breast cancer, HIV infections, osteoporosis, acute coronary syndrome, and they serve as immunosuppressants [8]. Several large peptide-based drugs include: cyclosporin A (MW = 1,185), caspofungin (MW = 1,093), vancomycin (MW = 1,431), and fuzeon (MW = 4,492). Cyclosporin A is an 11 amino acid macrocyclic peptide that is used to suppress the immune system after organ transplants [9]. Caspofungin, vancomycin, and fuzeon are peptide-based antifungal, antibacterial, and anti-HIV drugs, respectively. Aplidine (MW = 1,067) is an eight amino acid peptide-based cancer agent that is currently in clinical trials [10,11,12].

**Figure 1:**
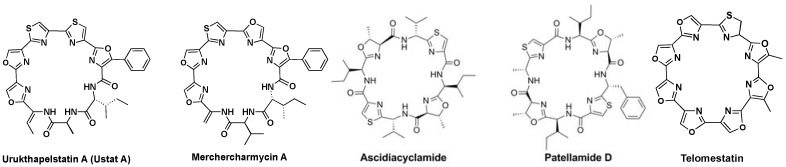
Ustat A and related structures: merchercharmycin A (IB-01211) [25,26,27], ascidiacyclamide [20], patellamide D, and telomestatin [25,28].

Urukthapelstatin A (Ustat A, Figure 1) falls in a unique class of natural products as it contains directly linked 4,2-azoles [13,14]. Over the last two decades there has been a surge in discoveries of natural products with directly linked azoles, many are now drug candidates [15,16,17]. Mixed 4,2-bisheterocycle tandem pairs are relatively rare in macrocycles, and thus, urukthapelstatin A represents a unique macrocyclic structure. Ustat A has an average GI_50_ of 5.6 nM on the Japanese 39 cell line panel [13,14], yet it’s mechanism of action remains unknown. As a modified macrocyclic octapeptide, Ustat A resembles several biologically active natural products including: merchercharmycin A (IB-01211), ascidiacyclamide, patellamide D, and telomestatin (Figure 1). IB-01211 was isolated from the marine microorganism strain ES7-008, and it is an antitumor macrocyclic peptide that has an IC_50_ = 14 nM in Hela S3 cells [18]. The same structure was proposed for merchercharmycin A, which was isolated from a marine-derived *Thermoactinomices sp.* and has an IC_50_ = 40 nM and 46 nM in A549 cells and Jurkat cells [19] respectively. Ascidiacyclamide is a cytotoxic cyclic peptide with thiazole and methyl-oxazoline rings [20] and it exhibits cytotoxic activity (IC_50_ = 14 µM) against leukemia cells [21]. Patellamide D that is a resistance-modifying agent, where it reverses resistance to the anti-cancer agent vinblastine [22]. Finally, the most investigated of these macrocycles is telomestatin. Telomestatin stabilizes the G-quadruplex found in telomeric DNA and inhibits telomerase activity (IC_50_ = 5 nM) in numerous cancerous cells, inducing apoptosis [23]. Telomestatin is currently in clinical trials [24]. The interesting biological activity displayed by these heterocycles appears related to their planar aromatic structures, which lend rigidity to the ring and provide sites for binding interactions via pi-stacking.

Depending on the natural product structure, using a solution phase approach can be advantageous over a solid-phase approach [29,30,31,32,33,34,35,36,37,38]. Synthesizing Ustat A in solution appeared desirable for several reasons. The first was that the syntheses of merchercharmycin A [25,26] were completed in solution, providing precedent. The second was the ability to explore whether the fragments of the heterocycles were cytotoxic [39]. Our experience and the complexity of Ustat A lead to our decision to initially explore a solution phase synthesis approach [40,41,42,43,44,45,46,47,48].

However, there are also numerous advantages to utilizing solid phase synthesis in building complex peptide-based macrocycles. Our expertise in solid phase synthesis of cyclic peptides has also been extensive [49,50,51]. General advantages of solid phase synthesis are easy purification, rapid generation of linear peptide intermediates, and precedent in the synthesis of large peptides. Given the ease with which peptide linear precursors are produced on solid phase, we opted to employ this approach in addition to a solution phase method. Should the solid-phase approach work, it would simplify analog synthesis. Thus, exploring the synthesis of the macrocycle Ustat A using both a solution phase and solid phase method provided opportunities to evaluate the benefits of both methods to synthesize this natural product. 

## 2. Results and Discussion

### 2.1. Synthetic Approach

**Scheme 1:**
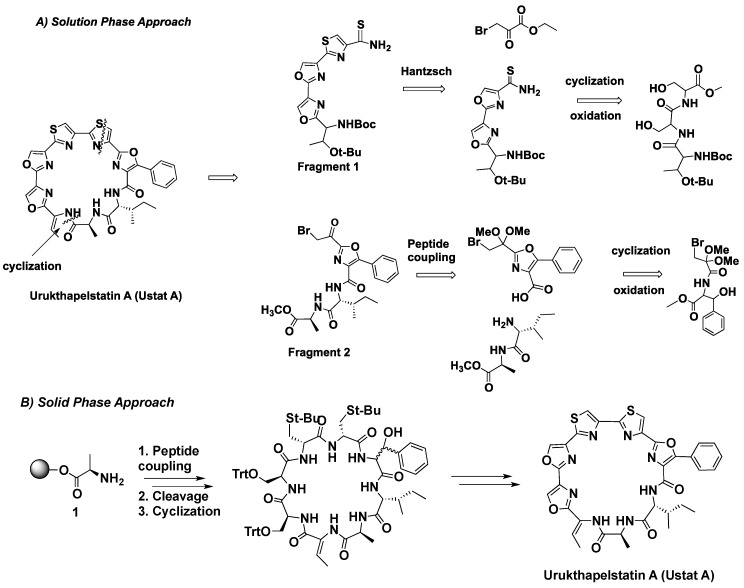
Two synthetic approaches: (**A**) solution phase and (**B**) solid phase synthesis of Ustat A.

Solution synthesis of Ustat A involved cyclization at the peptide bond after forming the linear heterocyclic peptide (Scheme 1A). Formation of the linear precursor involved a modified Hantzsch thiazole reaction between the thioamide and bromoketone (fragments 1 and 2 respectively). Synthesis of fragment 1 entailed cyclization and oxidation of serine residues into oxazoles and the subsequent modified Hantzsch thiazole reaction between ethyl bromopyruvate and the thioamide located on the dioxazole moiety (Scheme 1A). Solid phase synthesis started with an alanine residue attached to a chlorotrityl resin (Scheme 1B). Sequential coupling of residues, cleavage, and cyclization generates a peptide precursor. Subsequent cyclization and oxidation of the serines and cysteines to heterocycles would generate the desired natural product.

### 2.2. Solution Phase Synthesis

**Scheme 2:**
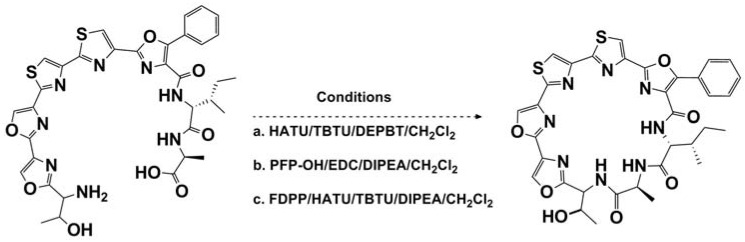
Attempted cyclization conditions for the formation of Ustat A.

We have published the formation of the Ustat A linear precursor that was generated in solution [52]. Several macrocyclization trials were performed from C- and N-deprotected linear precursor using these conditions with activating agents. Initially, cyclization of the macrocycle involved standard coupling conditions developed in our lab (Scheme 2, condition a) [42,50]. However, when these conditions were unsuccessful, we followed the conditions described by Hernandez *et al.* in the synthesis of merchercharmycin A [25,26,28] (Scheme 2, condition b). These conditions were also unsuccessful, and a closer analysis of Hernandez data on the cyclized product suggests that these conditions were also unsuccessful for synthesizing merchercharmycin A [28]. Additional cyclization conditions were attempted including esters activated by pentafluorophenyl diphenylphosphinate (FDPP) as reported by Joullié *et al.* (Scheme 2, condition c) [53]. All methods showed no trace of cyclized compound as determined by both LCMS and ^1^H-NMR. The unsuccessful cyclization of the linear heterocyclic Ustat A precursor using numerous conditions indicated that the sequential heterocycles created a molecule that was too rigid to cyclize with the heterocycles in place.

### 2.3. Solid Phase Synthesis

Synthesis using solid phase where construction of the heterocycles could be done after formation of the large macrocycle was appealing for three reasons. First, it would offer a simple option for substituting serines for cysteines allowing production of thiazoles in place of oxazoles. Given that thiazole fragments show promising biological activity, exchange of oxazoles for thiazoles is attractive [39]. Second, modification of the amino acids within the peptide region would be straightforward using solid phase, and analogs would be rapidly generated. Rapid production of these compounds provides the opportunity to explore structure activity relationships in biological systems. Third, forming the heterocycles after macrocycle formation allows the choice of sequentially closing the heterocycles, providing synthetic options for forming the final heterocyclic analogs.

**Scheme 3:**
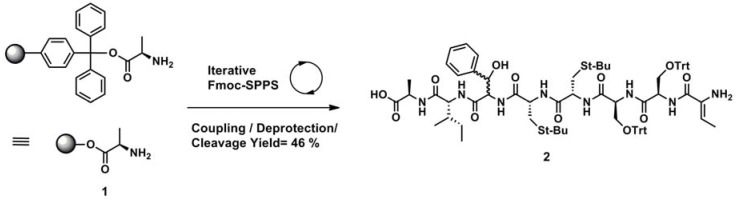
Synthesis of Ustat A using solid phase.

This is the first report of the solid phase synthesis of a Ustat A precursor. Starting with alanine bound to a chlorotrityl resin (**1**, Scheme 3), we sequentially coupled amino acids using *N*-hydroxy-benzotriazole (HOBt) and diisopropylcarbodiimide (DIC) in DMF and Fmoc removal using 20% piperidine and 80% DMF (Scheme 3) [50]. Upon generating the linear precursor on solid phase, cleavage to produce the desired compound **2** occurred using trifluoroethanol (TFE):CH_2_Cl_2_ (1:1, v/v) in a 46% yield. It was characterized by ^1^H-NMR, indicating we had successfully generated the linear peptide (Scheme 3).

**Scheme 4:**
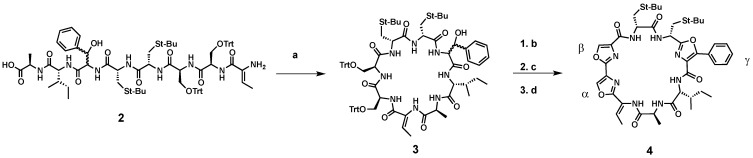
Cyclization of the linear precursor.

Cyclizing the linear precursor **2** utilizing our standard conditions [50] generated the cyclized compound **3** (Scheme 4, yield = 36%). Employing triethylsilane (TES):CH_2_Cl_2_ with 1.1% trifluoroacetic acid (TFA) in CH_2_Cl_2_ selectively removed the trityl protecting group (Trt) from serine in the presence of the ^t^Bu protecting groups on cysteines. Oxazoline formation was carried out by intramolecular cyclodehydration of serines using the fluorinating agent diethylamino sulfurtrifluoride (DAST) and pyridine/K_2_CO_3_ to afford all three intermediate oxazolines (yield = 47%). The oxazolines were oxidized using bromochloroform (BrCCl_3_) and 1,8-diazabicyclo[5,4,0]undec-7-ene (DBU) generating compound **4**.

## 3. Experimental

### 3.1. Materials

All reagents and solvents were commercially available and used without further purification. All reactions were carried out with dry solvents under argon. Silica gel 60 (230–400 mesh) was used for thin-layer chromatography (TLC), and flash chromatography. Reactions were monitored by LCMS (Shimadzu Liquid Chromatograph-Mass Spectrometry) or on TLC using UV light as visualizing method and KMnO_4_, Bromocresol Green, and Ninhydrin as developing agents. All amino acids are commercially available and solid phase reactions were monitored by the ninhydrin test. ^1^H- and ^13^C-NMR spectra were recorded with Bruker Avance III instruments operating at 300 MHz and 600 MHz, respectively. Accurate mass spectra for the novel molecules were recorded using a Thermo LTQ Orbitrap XL high resolution liquid chromatograph-mass spectrometer/mass spectrometer, equipped with a conventional ESI source.

### 3.2. Synthesis

#### 3.2.1. General Procedure for Solid Couplings

Solid-phase peptide synthesis was produced manually on 0.08 mmol scale in polypropylene peptide synthesis reaction vessels according to the following general procedures: pre-loaded *N*-deprotected resin was swelled by DMF for 30 min in the synthesis reaction vessel, drained, then subjected to the following coupling cycle for all standard Fmoc AA’s: Fmoc-protected amino acid (3 equiv. relative to resin loading) and HOBt (3 equiv.) dissolved in minimal DMF. DIC (6 equiv.) was added in one portion to resin slurry. The resulting solution was shaken for a minimum of 2 h. Some sample was removed from the resin and washed with IPA. It was checked by the ninhydrin test. Once the N-terminal amino acid residue has been coupled, the peptide-resin is washed with DMF (3 × 1 min). The resin was agitated in 20% piperidine/DMF (2 × 10 min), and then washed with DMF (3 × 1 min), IPA (3 × 1 min), and DMF (5 × 1 min). It was also checked by the ninhydrin test. 

#### 3.2.2. Solid Phase Synthesis of Linear Precursor **2**

Resin-bound linear precursor compound was generated following from alanine to (2*Z*)-2-amino-2-butenoic acid via the general procedure for solid coupling method (Section 3.2.1). Ordered Fmoc-AA’s: Resin based alanine, D-*allo*-isoleucine, racemic-phenyl serine, ^t^Bu-cysteine, ^t^Bu-cysteine, trityl-serine, trityl-serine, and (2*Z*)-2-amino-2-butenoic acid [55]. After the last Fmoc deprotection, the resin was washed with DMF (3 × 1 min), IPA (3 × 1 min) and MeOH (3 × 1 min). The linear peptide was cleaved from the resin by swelling and stirring the resin in a solution of TFE/CH_2_Cl_2_ (1:1, v/v, 10 mL/g of dried resin) for 24 h. Cleaved peptide solution was then filtered through a Buchner filter, drained resin was then washed with additional CH_2_Cl_2_ to fully extract cleaved peptide from resin. Solvents were then collected and removed by rotary evaporation and peptide was dried under high vacuum overnight. Dried peptide was then reconstituted with CH_2_Cl_2_ and evaporated multiple times by rotary evaporator before being dried *in vacuo* overnight to remove residual TFE to give a linear precursor as clear glassy solid to afford compound **2**. Yield: 46%; ^1^H-NMR (300 MH_Z_, Bruker, CDCl_3_), δ 7.51–7.08 (m, 35H), 5.46 (m, 1H), 4.73 (m, 1H), 4.58 (m, 1H), 4.58–4.32 (m, 4H), 4.0 (q, 4H, *J* = 8.31 Hz), 3.62–3.34 (m, 3H), 3.15 (q, 4H, *J* =7.20 Hz), 3.01–2.83 (m, 3H), 2.14 (m, 1H), 2.01–1.78 (bs, 2H), 1.68 (s, 3H), 1.40–1.25 (m, 18H), 1.05–1.02 (m, 3H), 0.87–0.85 (m, 3H); ^13^C-NMR (300 MHz, Bruker, CDCl_3_), δ 197.9, 172.1–169.5 (peptide amide C=O), 143.1, 128.4–126.3 (aromatic carbons, trityl), 126.1, 87,4, 72.4, 62.4, 60.4, 55.2, 54.7, 43.5 (peptide aliphatic carbons), 30.97 (^t^Bu-CH_3_), 25.9, 22.1, 17.6, 13.8, 11.3, 6.7 (peptide aliphatic carbons); LCMS (ESI): calcd for C_80_H_96_N_8_O_12_S_2_Na^+^ [M+Na^+^] 1448.78, found 1448.70; HRMS(ESI): calcd for C_80_H_9__7_N_8_O_12_S_2_ [M+H^+^] 1425.6667, found 1425.6443.

#### 3.2.3. Synthesis of the Cyclized Compound **3**

The cyclized compound **3** was synthesized using linear compound **2** (0.8 g) in CH_2_Cl_2_ (280 mL, 2 mM) with HATU (0.10 g, 0.5 equiv.), TBTU (0.09 g, 0.5 equiv.), DMTMM (0.07 g, 0.5 equiv.), COMU (0.11 g, 0.5 equiv.) as the coupling reagents with anhydrous DIPEA (0.78 mL, 8 equiv.). The reaction mixture was stirred at room temperature for 6 h. Upon completion, the reaction was washed with aqueous HCl solution (pH = 1, 50 mL × 2), and saturated aqueous NaHCO_3_ solution (50 mL × 2). The collected organic layer was dried over Na_2_SO_4_ and concentrated *in vacuo*. The obtained crude residue was purified by flash column chromatography (silica gel, EtOAc/hexanes) to yield the desired cyclized compound **3** as a yellow oil (28 mg). The cyclization reaction to afford compound **3** was found to be rapid and efficient by LCMS analysis. Yield: 36%; ^1^H-NMR (300 MHz, CDCl_3_,) δ 7.51–7.10 (m, 35H), 5.40 (m, 1H), 4.81 (m, 1H), 4.70 (m, 1H), 4.36–4.21 (m, 4H), 4.20–3.91 (m, 4H), 3.56–3.34 (m, 3H), 3.17–2.86 (m, 7H), 2.38–2.33 (m, 1H), 2.01 (s, 3H), 1.62 (m, 2H), 1.40–1.25 (m, 18H), 0.93–0.88 (m, 6H); ^13^C-NMR (600 MHz, Bruker, CDCl_3_), δ 176.8–171.2 (peptide amide C=O), 146.8, 131.6–125.7 (aromatic carbons, trityl), 120.5, 115.1, 66.6, 60.4, 56.9, 54.4, 48.8, 43.9, 36.7, 32.6 (peptide aliphatic carbons), 29.4 (^t^Bu-CH_3_), 27.1, 25.2, 22.7, 20.6, 14.2 (peptide aliphatic carbons); LCMS (ESI): calcd for C_80_H_95_N_8_O_11_S_2_ [M+H^+^] 1408.78, found 1408.95; HRMS(ESI): calcd for C_80_H_9__4_N_8_O_1__1_S_2_Na [M+Na] 1429.6381, found 1429.6691.

#### 3.2.4. Synthesis of Oxazole-based Macrocycle **4**

Selective removal of Trts: compound **3** (0.28 g) was diluted to a 0.1 M solution in TES/CH_2_Cl_2_ (1:1, v/v), followed by the addition of 1.1% TFA in CH_2_Cl_2_ until colorless. The reaction was performed at room temperature for 3 h. Upon completion, the reaction mixture was dried, and concentrated to afford the alcohol (quantitative yield).

Oxazoline formation: 3.5 equiv. of DAST (0.14 mL) were added dropwise to the serine macrocyclic compound in CH_2_Cl_2_ (3.0 mL, 0.1 M) at −70 °C under Ar. The reaction mixture was stirred for 1 h, followed by the addition of pyridine (3 equiv., 0.07 mL), and K_2_CO_3_ (1.5 equiv., 0.06 g) and stirred for an additional 30 min. The solution was then allowed to room temperature for 12 h. Upon completion, the organic solution was partitioned between saturated aqueous NaHCO_3_ and CH_2_Cl_2_. The solvent was removed and the residue was purified by flash column chromatography.

Oxazole formation: Oxidation of the oxazoline fragment was performed with DBU (6 equiv., 0.29 mL) in CH_2_Cl_2_ at −40 °C. The solution was stirred for 15 min and BrCCl_3_ (6 equiv., 0.21 mL) were then added to the reaction mixture, which was allowed to proceed over 12 h. The reaction was worked up using aqueous HCl solution (pH = 1, 50 mL × 2), and saturated aqueous NaHCO_3_ solution (50 mL × 2). The solvent was removed and the residue was purified by flash column chromatography to afford compound **4**. Yield: 56% (2 step); ^1^H-NMR (600 MHz, CDCl_3_), δ 7.38–7.24 (m, 5H), 7.19–7.17 (d, 2H, *J* = 7.48 Hz), 5.38 (m, 1H), 4.39–4.18 (m, 2H), 4.17–4.06 (m, 2H), 4.05–3.91 (m, 4H), 2.36 (s, 3H), 2.06 (m, 1H), 1.80–1.61 (m, 3H), 1.50 (m, 2H), 1.40–1.25 (m, 18H), 0.93–0.88 (m, 6H); ^13^C-NMR (600 MHz, Bruker, CDCl_3_), δ 173.5 (C=O), 165.9 (C=O), 146.8, 143.9 (oxazoles-CH), 134.2, 129.5, 129.4–126.3 (aromatic carbons, trityl), 68.1, 67.7, 56.8, 38.9, 34.5, 31.4 (peptide aliphatic carbons), 29.7 (^t^Bu-CH_3_), 28.9, 24.5, 23.8, 14.1, 11.0 (peptide aliphatic carbons); LCMS (ESI): calcd for C_42_H_54_N_8_O_8_S_2_Na^+^ [M+Na^+^] 886.05, found 886.10; HRMS(ESI): calcd for C_42_H_55_N_8_O_8_S_2_ [M+H^+^] 863.3584, found 863.3579.

## 4. Conclusions

We have developed a solid phase method for generating the potential anticancer lead Ustat A. Solid phase is superior to solution phase as it rapidly generates a flexible linear precursor. The linear peptide is easily cyclized (yield = 36%) and conversion of the serines and phenylserine to oxazoles is straightforward. Formation of the thiazoles remains, and synthetic studies to this end are ongoing. In addition, biological evaluation of intermediates **2**–**4** are in progress and will be reported in due course.

## Figures and Tables

*Reagents and conditions*: (**a**) *O*-(Benzotriazol-1-yl)-*N,N,N′,N′*-tetramethyluronium tetrafluoroborate (TBTU, 0.5 equiv.), 2-(1*H*-7-azabenzotriazol-1-yl)-1,1,3,3-tetramethyluronium hexafluorophosphate methanaminium (HATU, 0.7 equiv.), 3-(diethoxyphosphoryloxy)-3*H*-benzo[d][1,2,3] triazin-4-one (DEPBT, 0.5 equiv.), *N,N*-Diisopropylethylamine (DIPEA, 8 equiv.) in DCM (0.01 M); (**b**) Pentafluoropropanol (2 equiv.), 1-ethyl-3-(3-dimethylaminopropyl)carbodiimide (EDC, 2.5 equiv.), DIPEA (6 equiv.) in DCM (0.7 mM); (**c**) FDPP (2 equiv.), HATU (0.6 equiv.), TBTU (0.6 equiv.), DIPEA (6 equiv.) in DCM (0.7 mM).

*Reagents and conditions*: (coupling) Fmoc-Xaa-OH (3 equiv.), HOBt (3 equiv.), DIC (6 equiv.), DMF; (deprotection) 20% piperidine in DMF; (cleavage) TFE/CH_2_Cl_2_ (1:1, v/v).

*Reagents and conditions*: (a) HATU (0.5 equiv.), TBTU (0.5 equiv.), ethyl 2-cyano-2-(hydroxyimino)acetate (COMU, 0.5 equiv.), 4-(4,6-dimethoxy-1,3,5-triazin-2-yl)-4-methyl morpholinium chloride (DMTMM, 0.5 equiv.), DIPEA (8 equiv.) in CH_2_Cl_2_ (0.002 M); (b) TES/CH_2_Cl_2_ (1:1, v/v), 1.1% TFA in CH_2_Cl_2_ [54]; (c) DAST (3.5 equiv.), pyridine (3 equiv.), K_2_CO_3_ (1.5 equiv.) in CH_2_Cl_2_ (0.1 M), −70 °C; (d) DBU (6 equiv.), BrCCl_3_(6 equiv.) in CH_2_Cl_2_ (0.1 M), −40 °C.

## Supplementary Materials

Supplementary materials can be accessed at: http://www.mdpi.com/1420-3049/18/1/1111/s1.
