# Supplementary material for: Solid Phase versus Solution Phase Synthesis of Heterocyclic Macrocycles
Source: Molecules. 2013 Jan 16;18(1):1111–21. doi: 10.3390/molecules18011111 (PMC6269862; doi:10.3390/molecules18011111)

Solid Phase *versus* Solution Phase Synthesis of Heterocyclic Macrocycles

Seong Jong Kim[1] and Shelli R. McAlpine[1,*]

[1] School of Chemistry, University of New South Wales, Sydney, NSW 2052 Australia

E-Mail: s.mcalpine@unsw.edu.au (S.R. McAlpine)

Supporting Information Available

Spectra of compounds

Linier precursor (**2**)……………………………………………………………….…..S2

LCMS (ESI)

HRMS (ESI)

^{1}H NMR

^{13}C NMR

Cyclized compound (**3**)…..…………………………………………………….………….S6

LCMS (ESI)

HRMS (ESI)

^{1}H NMR

^{13}C NMR

Oxazoles contained macrocycle (**4**)…………………..…………………………….………S10

LCMS (ESI)

HRMS (ESI)

^{1}H NMR

^{13}C NMR

Linier precursor (**2**)_LCMS(ESI)

==== Shimadzu LCMSsolution Analysis Report ====

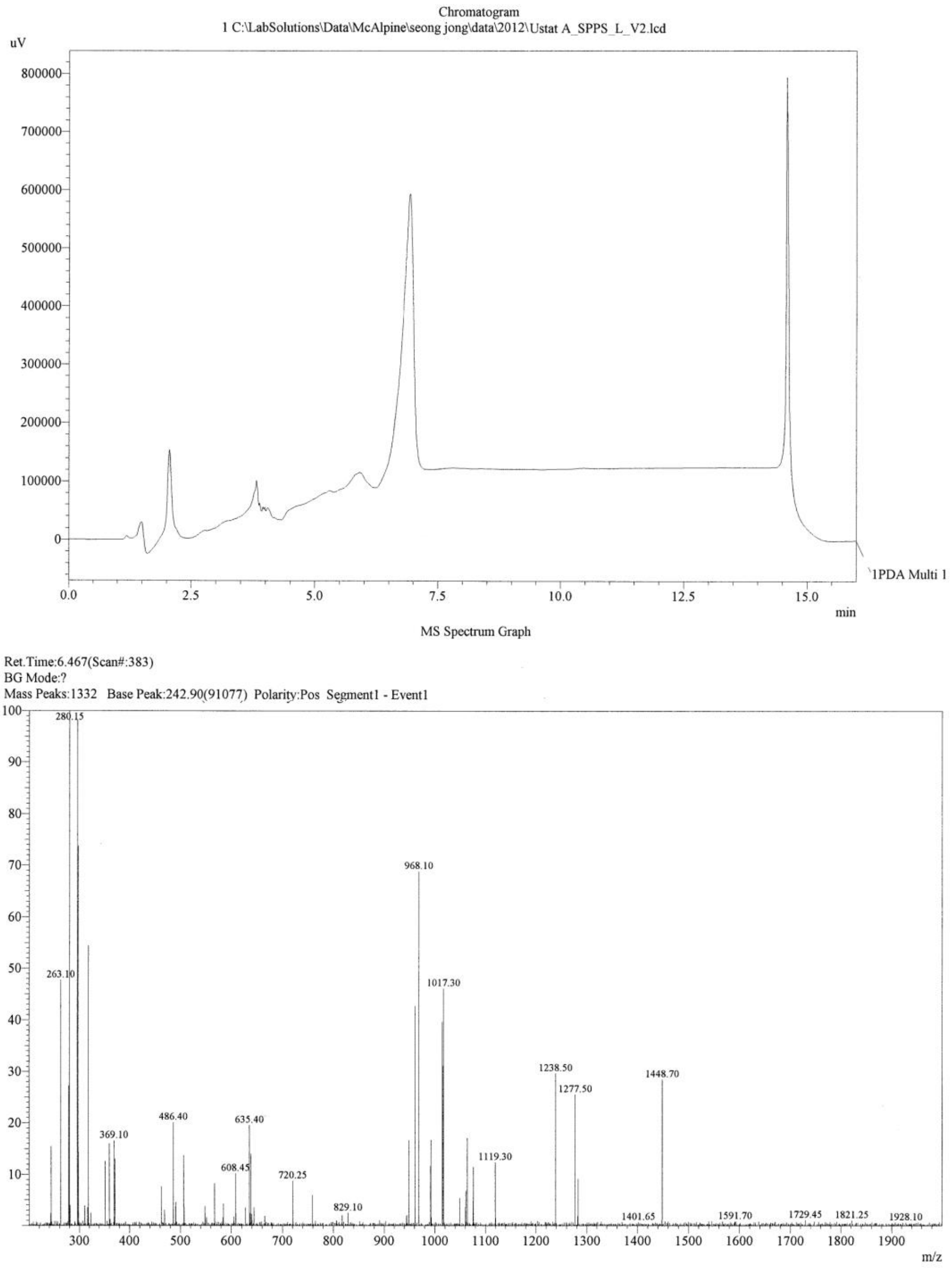

Linier precursor (**2**)_HRMS (ESI)

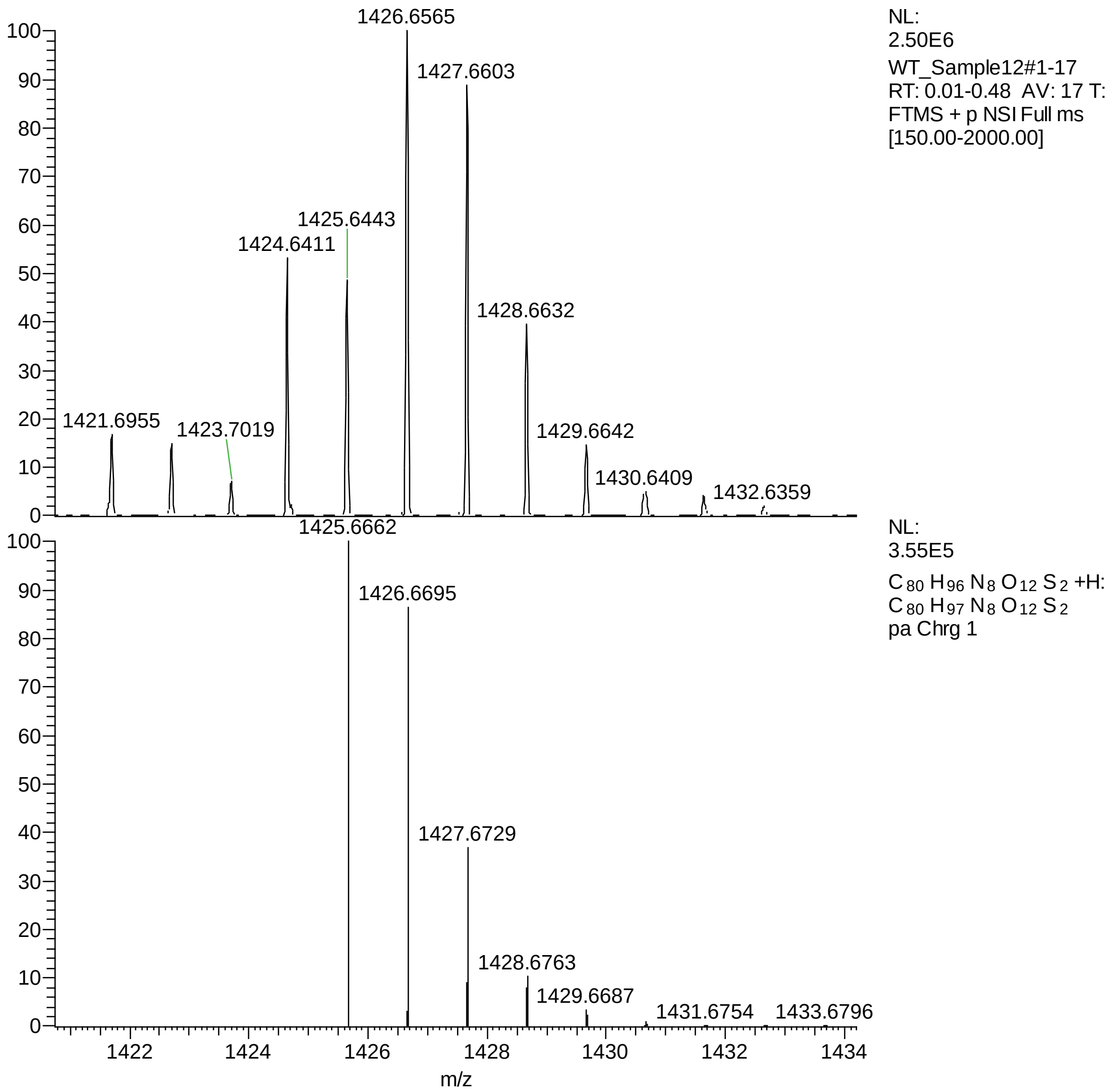

Linier precursor(**2**) _ ^{1}H NMR

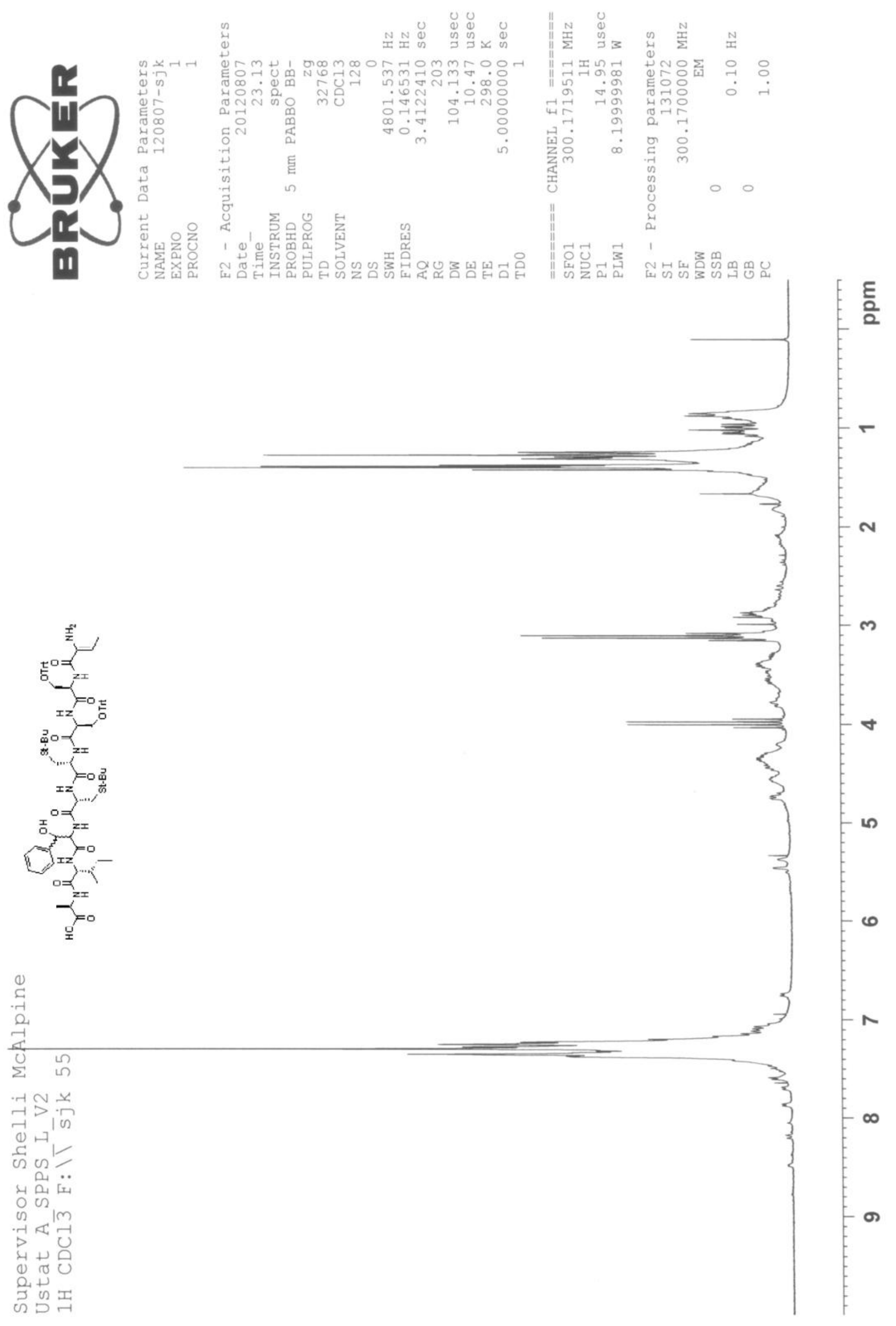

Linier precursor(**2**)_^{13}C NMR

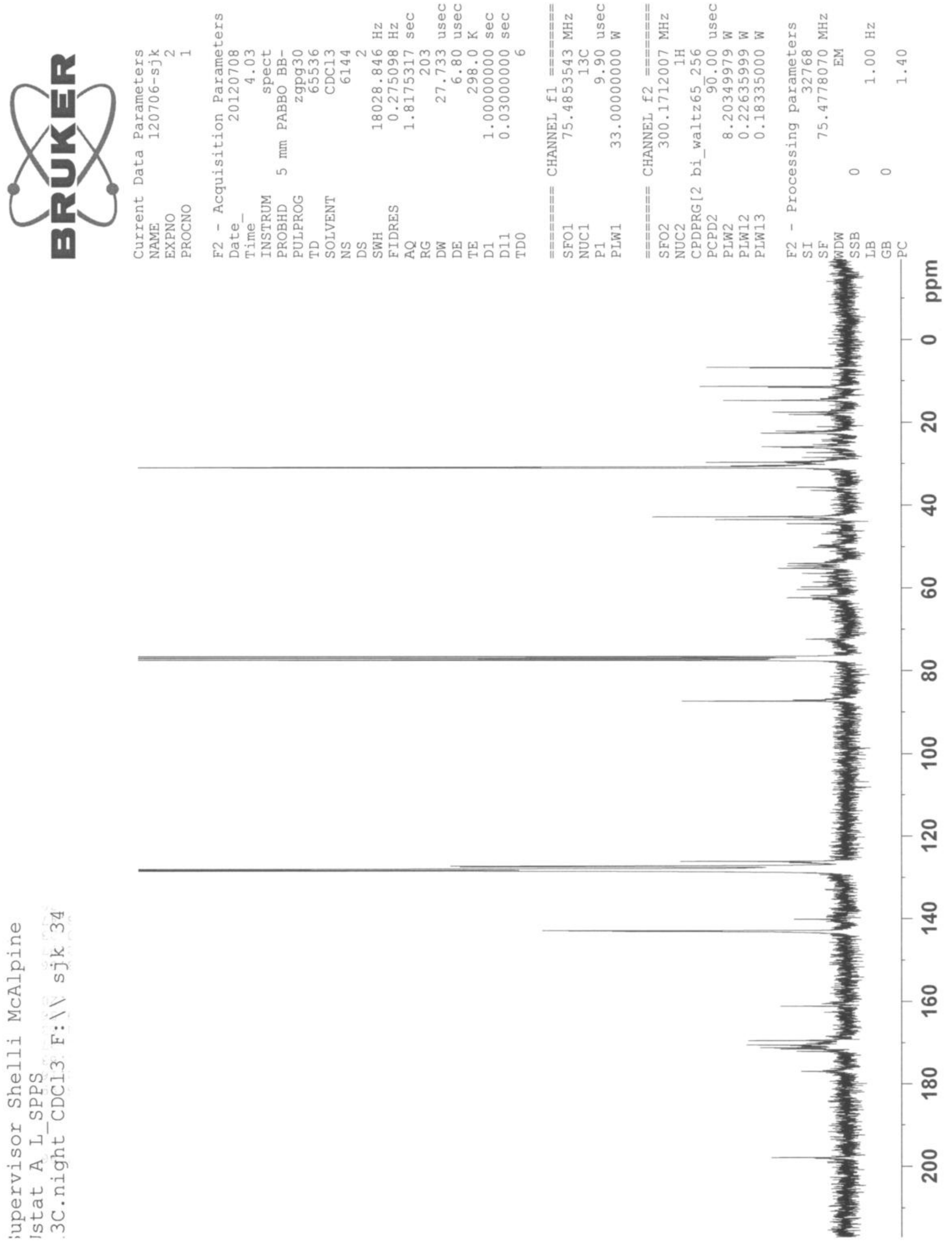

Cyclized compound(**3**)_LCMS (ESI)

==== Shimadzu LCMSsolution Analysis Report ====

Chromatogram
1 C:\LabSolutions\Data\McAlpine\seong jong\data\2012\UA_SPPS_CYC_SPOT 3.lcd

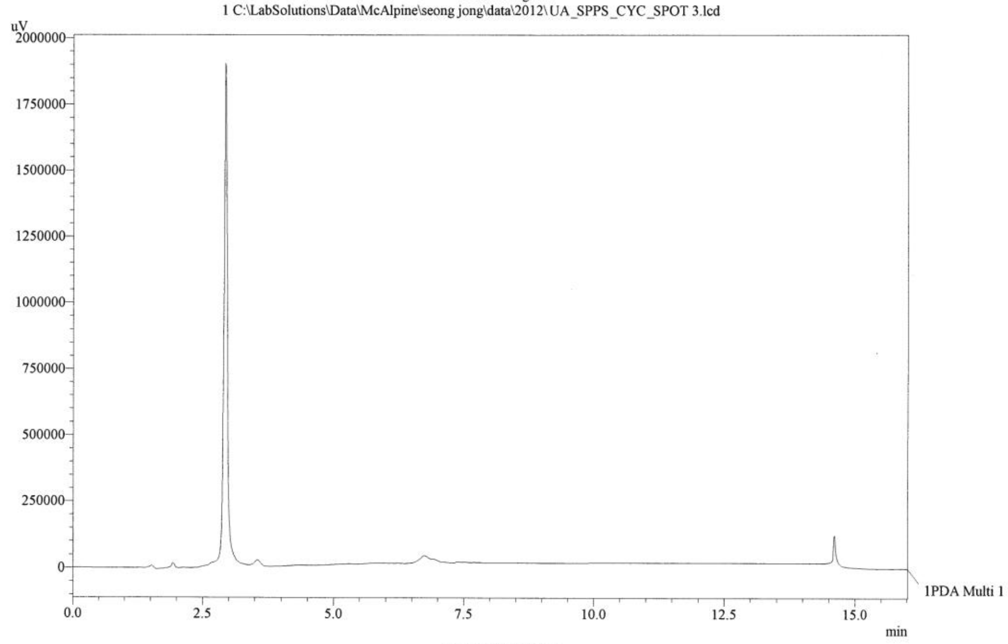

MS Spectrum Graph

Ret.Time:3.100(Scan#:181)
BG Mode:?
Mass Peaks:1338 Base Peak:274.90(60757) Polarity:Pos Segment1 - Event1

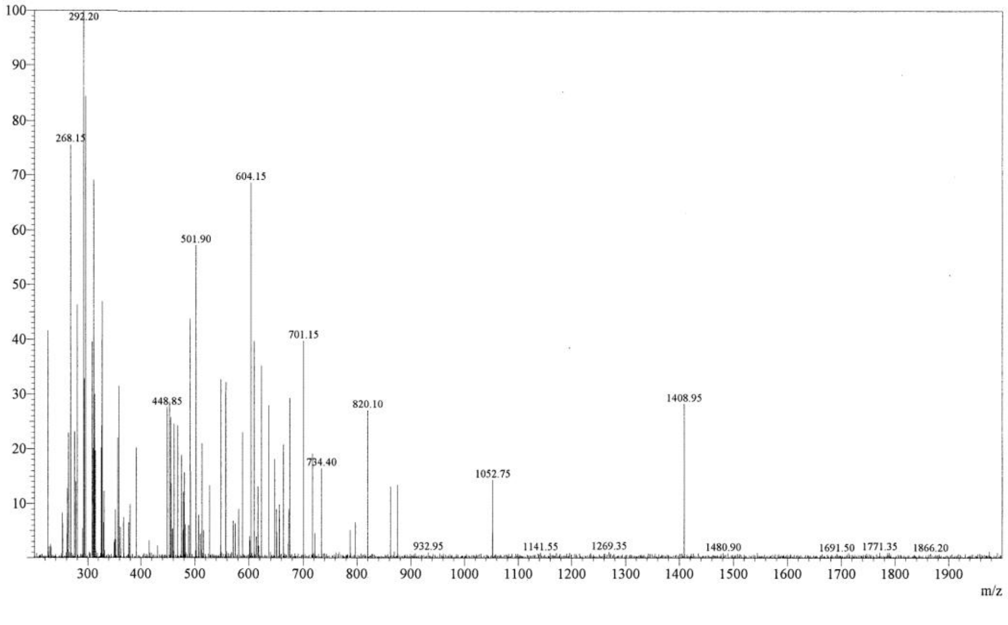

Cyclized compound(**3**) _ HRMS (ESI)

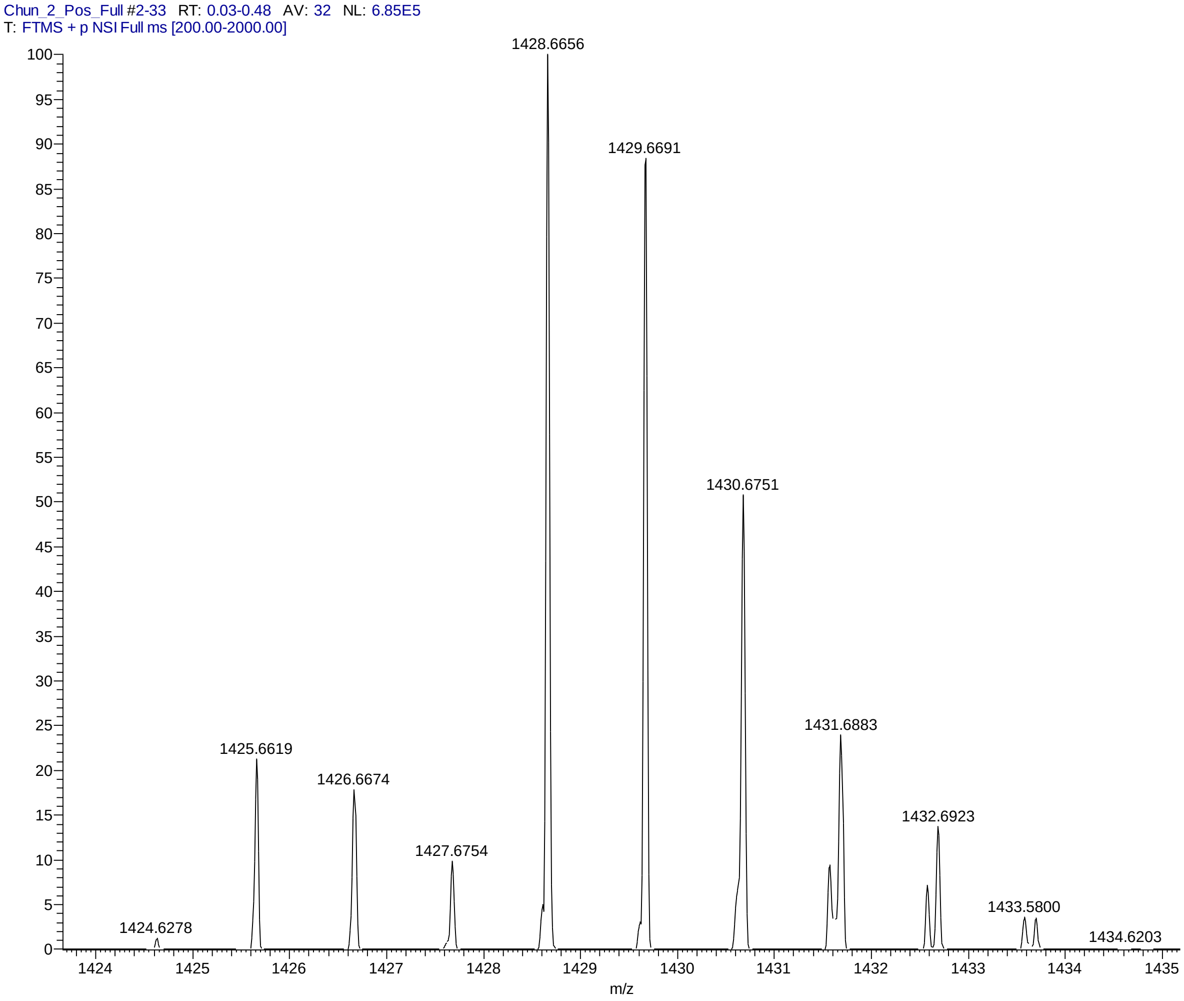

Cyclized compound(**3**) _^{1}H NMR

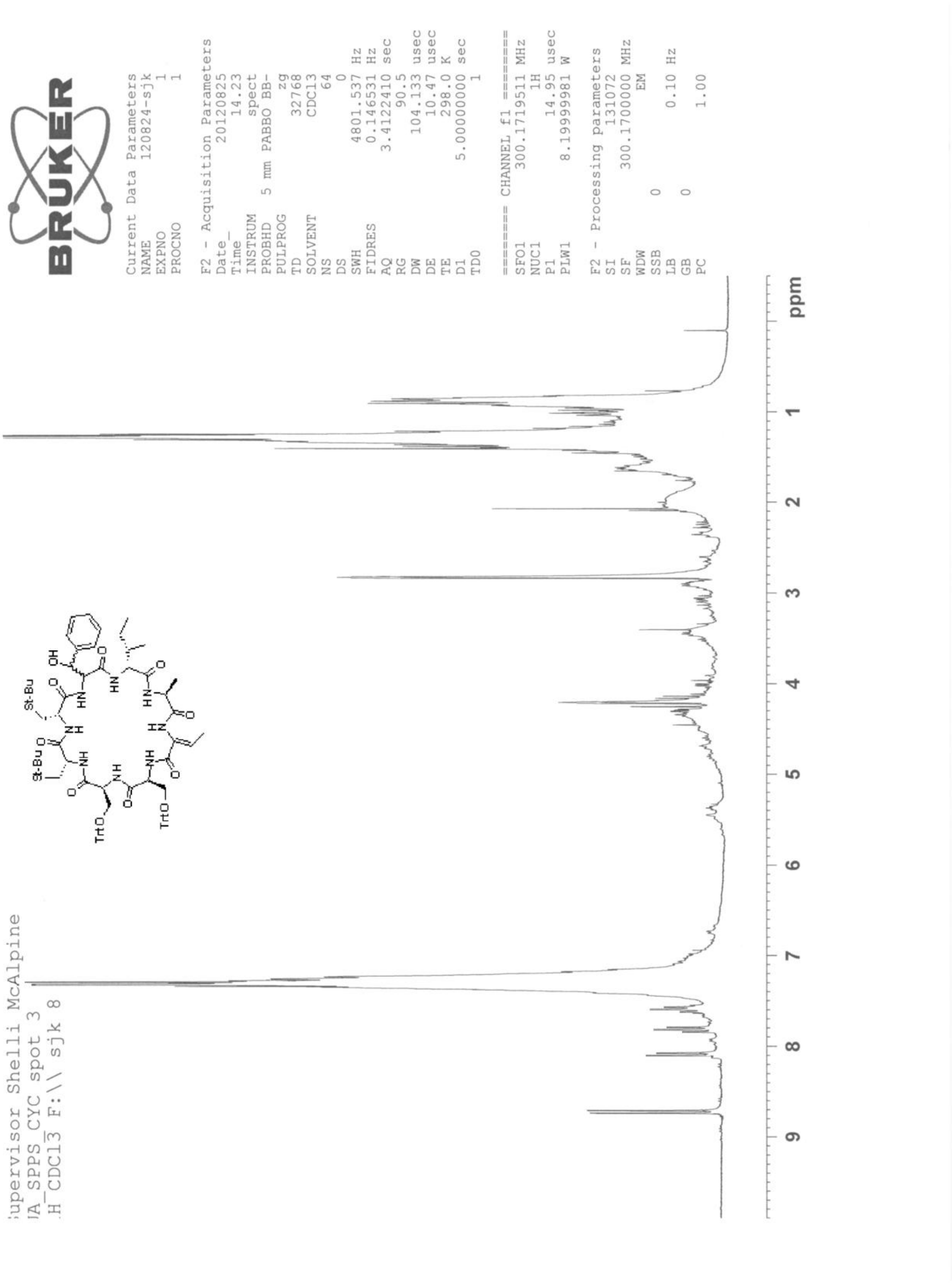

Cyclized compound(**3**) _ ^{13}C NMR

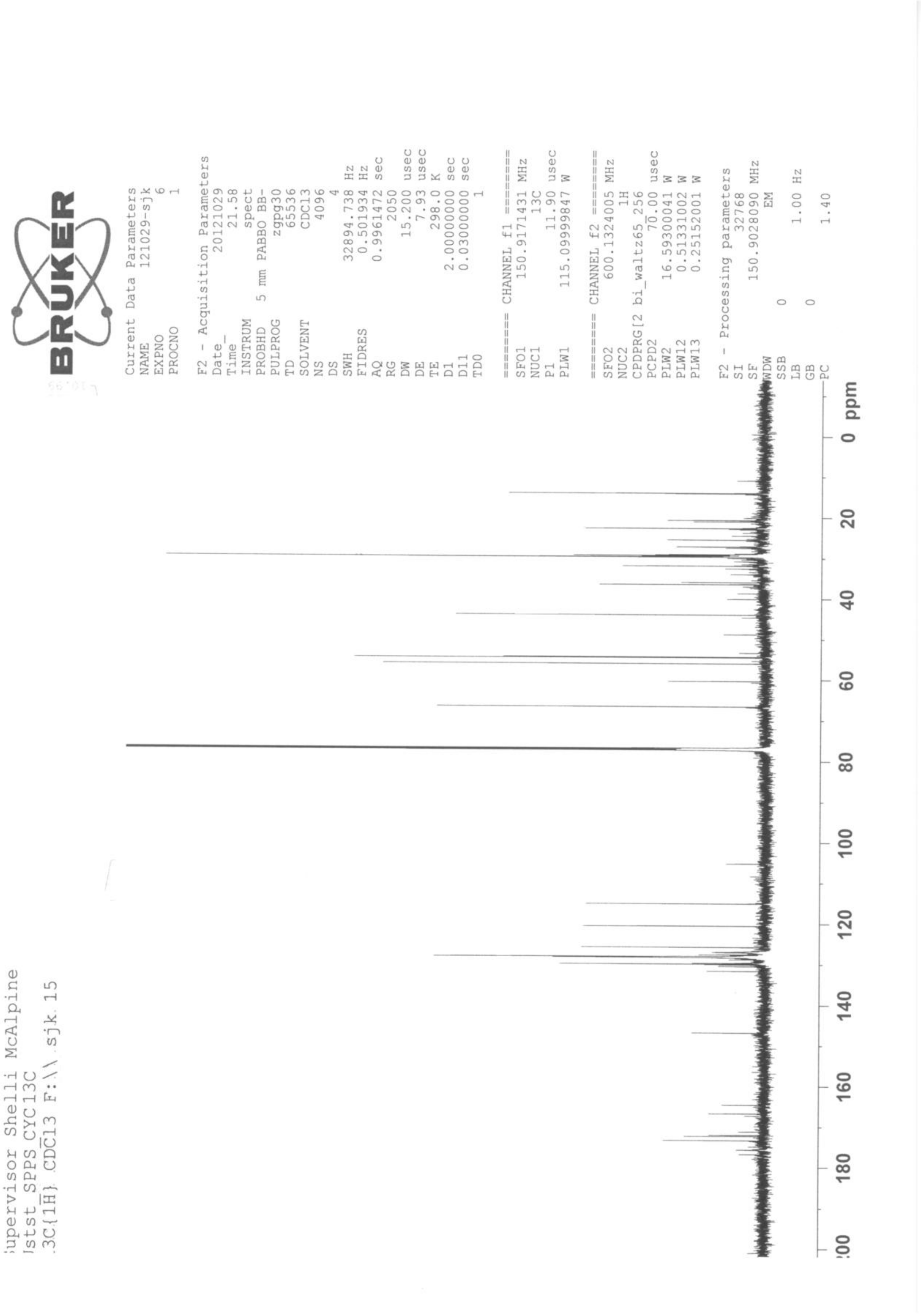

Oxazoles contained macrocycle (**4**)_LCMS (ESI)

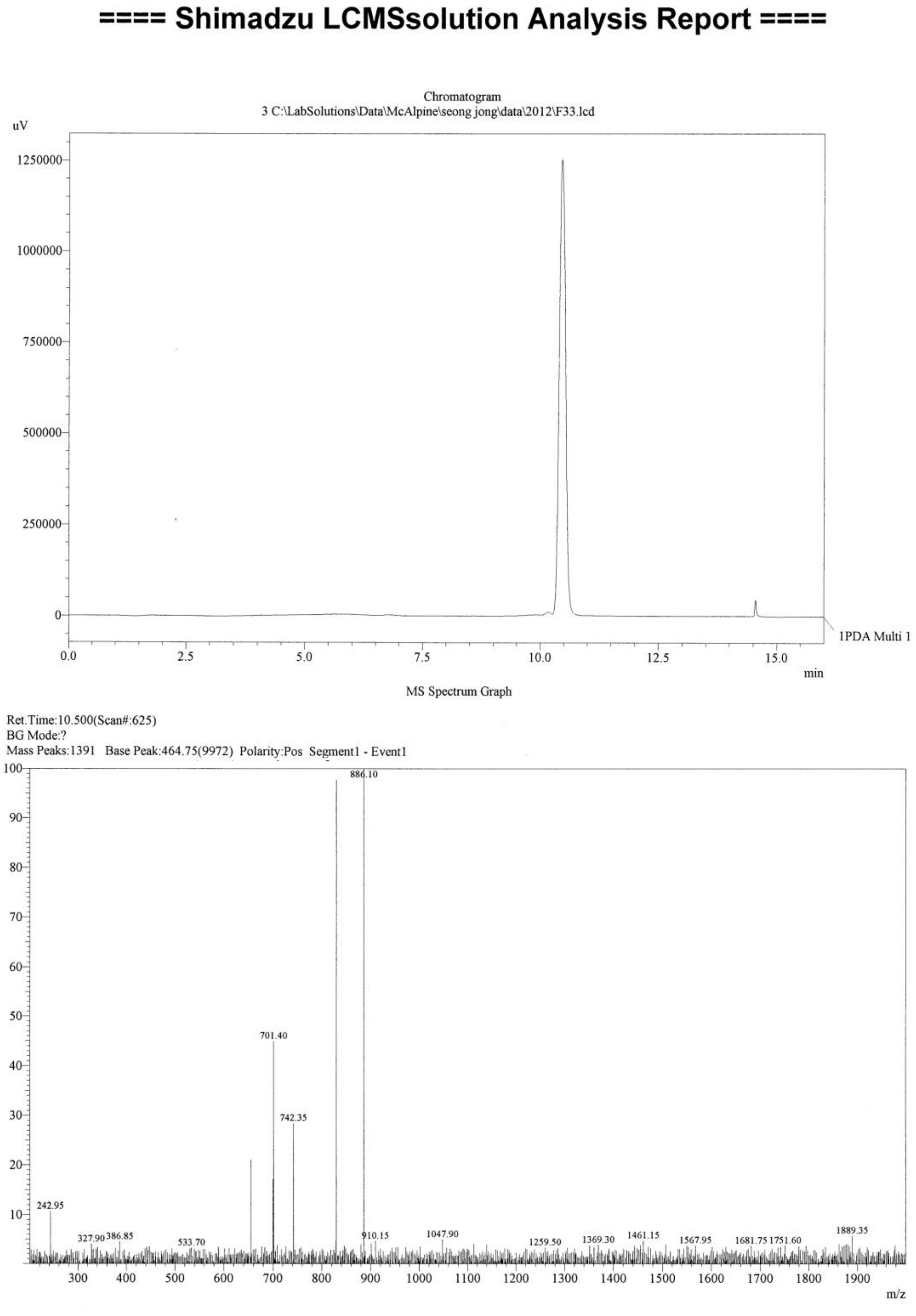

Oxazoles contained macrocycle (**4**) _ HRMS (ESI)

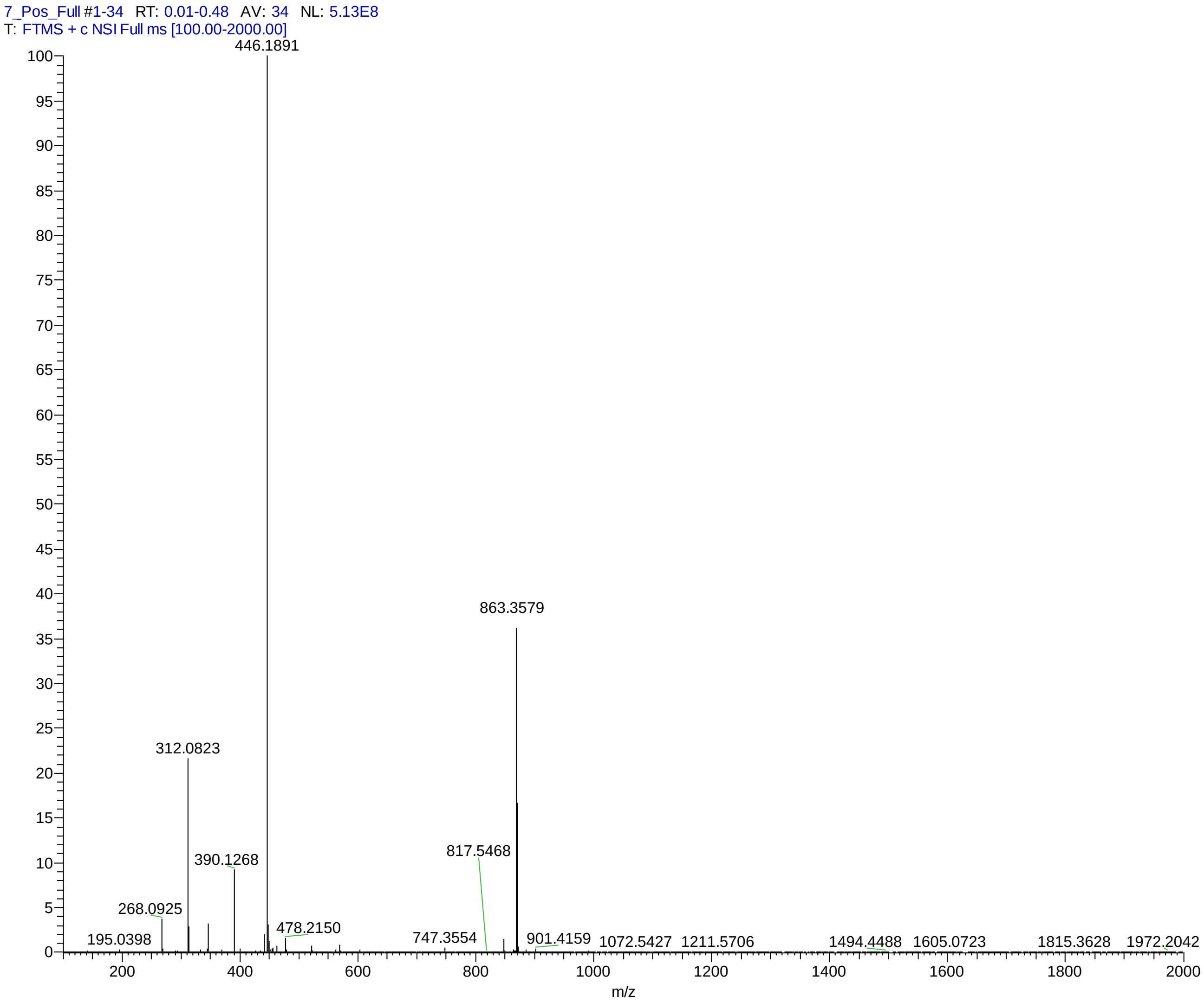

Oxazoles contained macrocycle (**4**)_^{1}H NMR

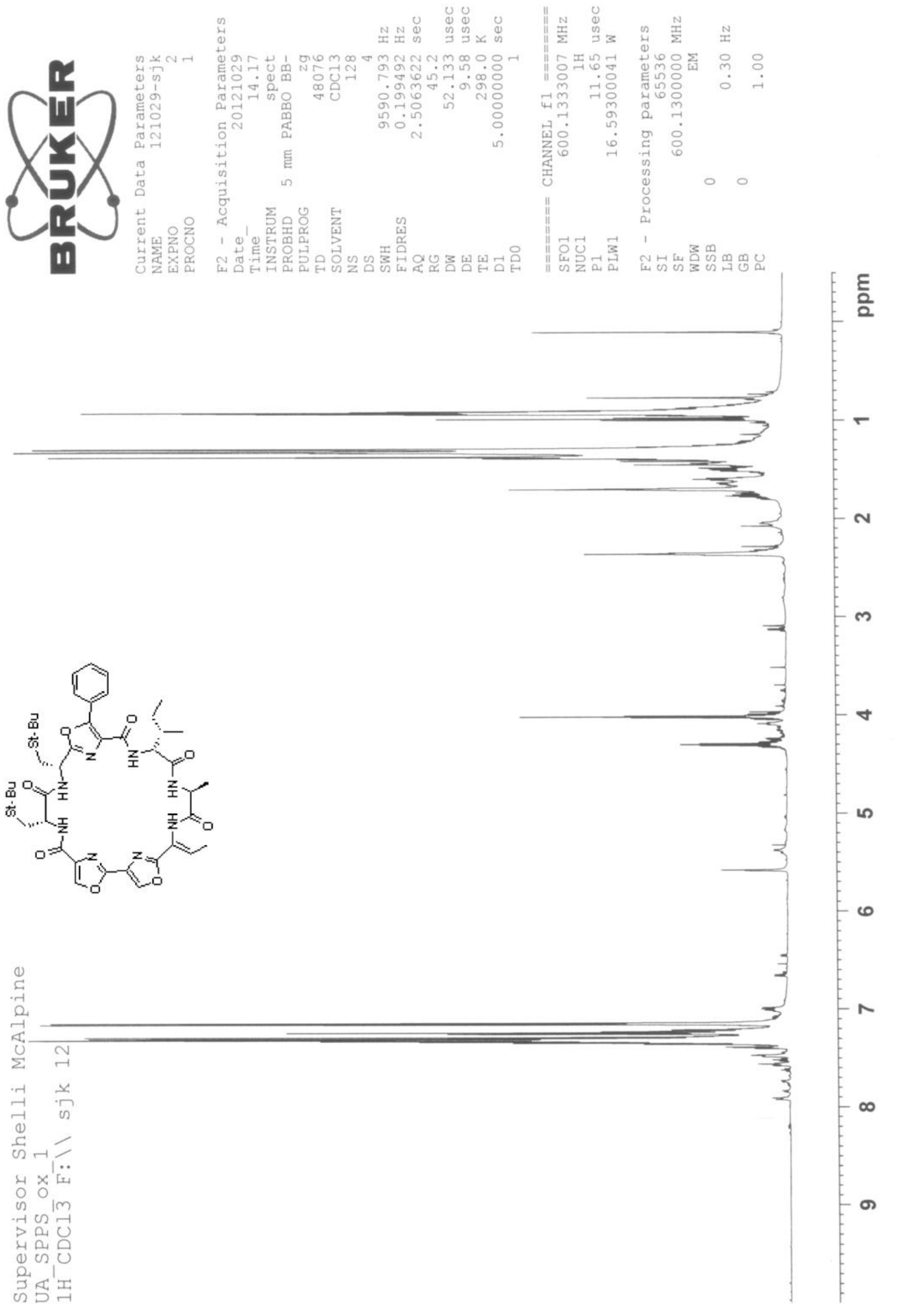

Oxazoles contained macrocycle (**4**)_^{13}C NMR

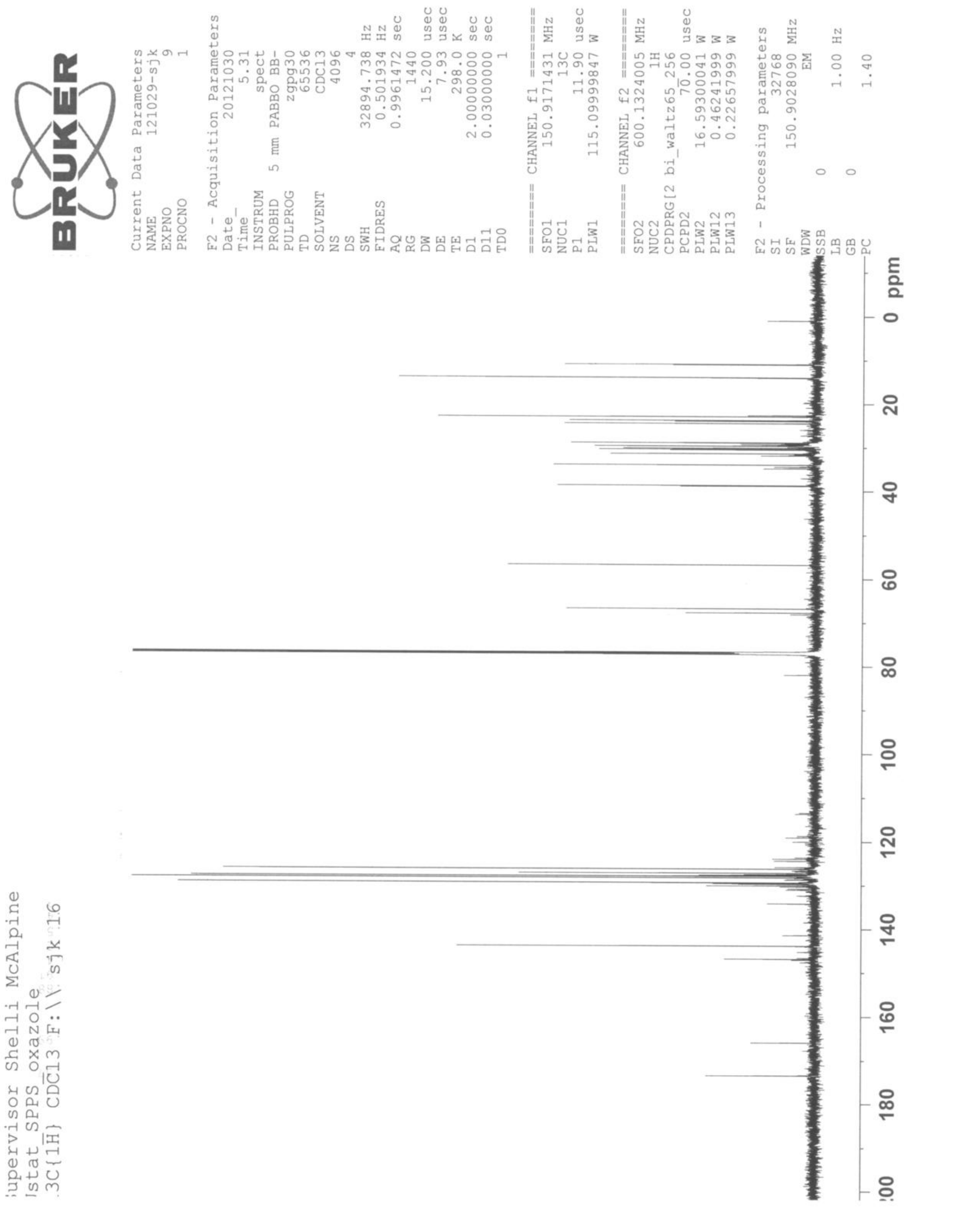

Supplement: Supplementary file 1 [file molecules-18-01111-s001.pdf]
